# Cryopreserved venous allograft in the treatment of a mycotic abdominal aortic aneurysm caused by group B Streptococcus

**DOI:** 10.1016/j.jvscit.2021.10.014

**Published:** 2021-11-17

**Authors:** Tyler D. Yan, Gary K. Yang

**Affiliations:** a Faculty of Medicine, University of British Columbia, Vancouver, British Columbia; b Division of Vascular Surgery, University of British Columbia – Okanagan, Kelowna, British Columbia

**Keywords:** Group B Streptococcus, *Streptococcus agalactiae*, Cryopreserved femoral vein, Mycotic aortic aneurysm, Cryopreserved venous allograft

## Abstract

We report a case of a mycotic abdominal aortic aneurysm caused by invasive group B streptococcus. Given the anatomical suitability with healthy segments of aortoiliac vessels, in situ repair was performed. A cryopreserved femoral vein graft was chosen because of risks of graft reinfection and negated the need for bilateral femoral vein harvest. The patient remained clinically well and the graft patent with no concerns at 6 months of follow-up. A review of literature on group B Streptococcus aortitis was performed.

Mycotic aneurysms account for less than 2% of surgically repaired aortic aneurysms but are nevertheless associated with marked morbidity and a mortality rate of 15%-50%.^1^, ^2^, ^3^ The management of mycotic abdominal aortic aneurysms (AAAs) includes antibiotics and timely surgical repair. The decision between ex situ and in situ repair depends on the patient stability, comorbidity, anatomy, and organism virulence. With improved conduits and antibiotic coverage, in situ repair has gained popularity, often involving grafts such as rifampin-impregnated prosthetics, cryopreserved arterial allografts, or autogenous vein grafts.^4^

The predominant causative organisms include *Salmonella* and *Staphylococcus*, and less commonly *Klebsiella, Campylobacter*, *Escherichia coli*, group A Streptococcus, *Enterococcus*, *Treponema pallidum*, *Mycobacterium tuberculosis,* or fungi (particularly *Candida* or *Aspergillus*).^5^ Group B Streptococcus (GBS) is a common inhabitant of the gastrointestinal and genitourinary systems and can cause a wide spectrum of invasive disease, but is rarely reported among mycotic aneurysms.^6^ We report a case of infectious aortitis caused by GBS and repaired with a cryopreserved femoral vein allograft. Patient consent was obtained for this case report.

## Case presentation

A 70-year-old woman presented with a 1-week history of nonspecific lower abdominal pain bilaterally. Comorbidities included type 2 diabetes mellitus, obstructive sleep apnea, and dyslipidemia. She denied any fever or chills, ill contacts, recent dental procedures, or other recent infections. Her white blood cell count was elevated at 13.7 × 10^9^/L and C-reactive protein was mildly elevated at 11.5 mg/L. All other laboratory workups were otherwise unremarkable. She had an abdominal/pelvis computed tomography (CT) scan that showed nonspecific fat stranding around a mildly dilated aorta maximally measuring 3.0 × 2.7 cm (Fig). She was empirically treated with piperacillin/tazobactam for presumed diverticulitis.

**Fig:**
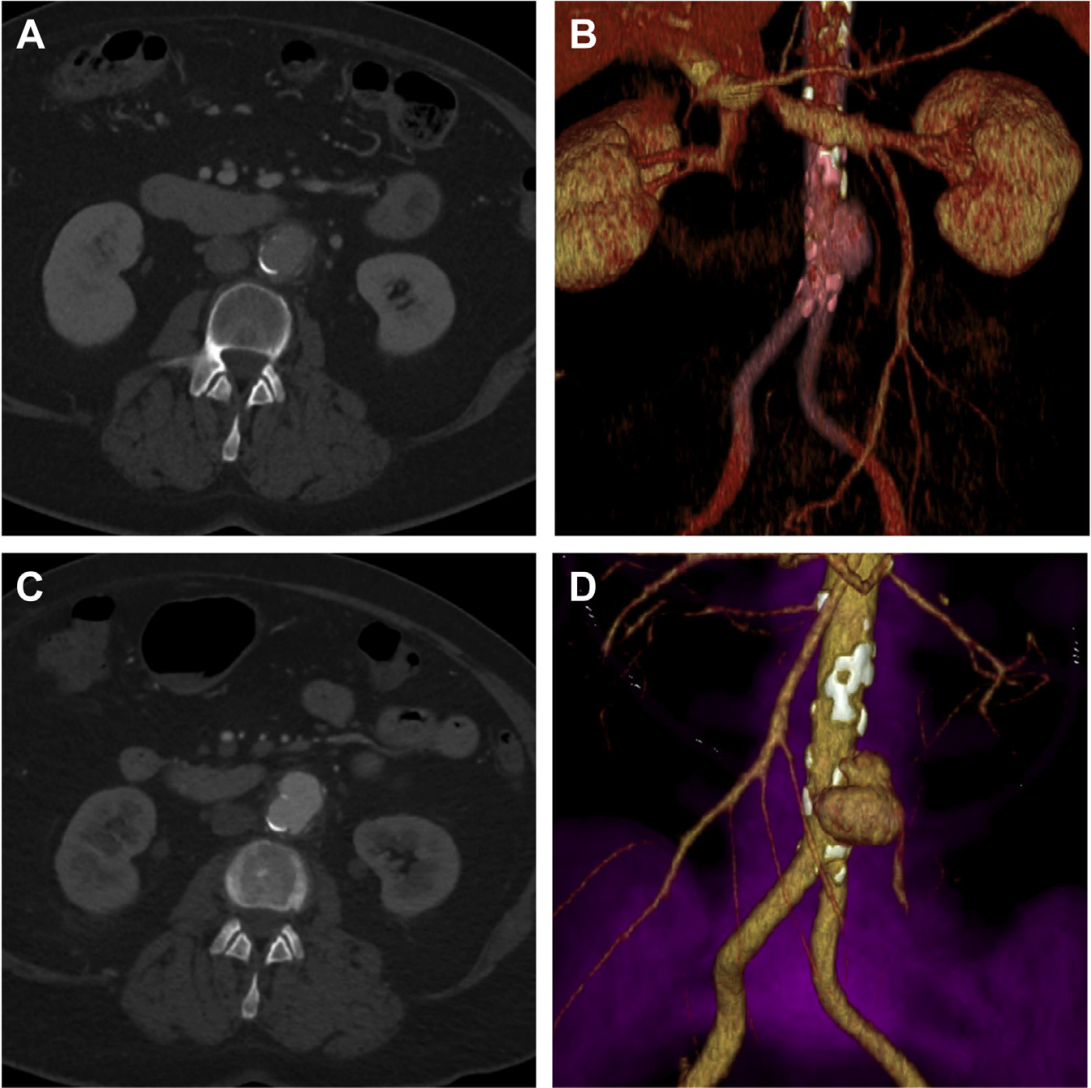
Preoperative computed tomography (CT) imaging. **A,** Initial CT scan with contrast demonstrating a mildly dilated aorta with a maximal diameter of 3.0 × 2.7 cm and localized inflammatory changes. **B,** Corresponding volume rendering image of the aortic aneurysm. **C,** Subsequent CT angiogram (CTA) 2 days later showing an increase in maximal measurement to 3.6 × 3.0 cm in addition to a penetrating ulcer and para-aortic stranding. **D,** 3D reconstruction of the CTA displaying the enlarging aneurysm.

However, a CT angiogram 2 days later demonstrated interval aneurysmal dilation to 3.6 × 3.0 cm and an underlying penetrating ulcer (Fig). This was now presumed to be mycotic in nature and definitive surgical repair was warranted. Given the patient’s stability and anatomic suitability, the decision was made for in situ reconstruction with a cryopreserved femoral vein graft (Cryolife).

The patient was brought to the operating theatre in stable condition. A standard midline laparotomy did not reveal any significant intraperitoneal fluid. There was significant retroperitoneal inflammation and tissue adhesion. No hematoma or other signs of rupture were encountered. No purulent discharge was noted. The infected aorta was excised and a tissue biopsy was sent for culture. The cryopreserved femoral vein was transected into two pieces and then cut longitudinally to create a larger panel graft. This was sewn together with 5-0 Prolene sutures with two continuous suture lines. The graft was anastomosed into the aorta using double-armed 3-0 Prolene sutures to completion.

The patient remained stable and her pain completely resolved after the procedure. The surgical tissue culture surprisingly grew GBS (*Streptococcus agalactiae*). Blood cultures remained negative after 5 days. Infectious diseases was consulted, and the patient was switched to ceftriaxone for 6 weeks, followed by oral amoxicillin for a year thereafter. The patient was discharged 10 days postoperatively. At 6 months of follow-up, she remained clinically well and CT imaging demonstrated resolution of surrounding stranding without any signs of pseudoaneurysm or deformities.

## Discussion

GBS is a gram-positive, facultative anaerobic bacteria often implicated in skin and soft-tissue infections, bacteremia without a focus, endocarditis, osteomyelitis, neonatal meningitis, urinary tract infections, and pneumonia.^7^ A mycotic AAA due to GBS has been rarely documented and was first reported in 1989 in the United Kingdom.^8^ In total, there have been nine previously reported cases of mycotic AAAs due to GBS infection.^8^, ^9^, ^10^, ^11^, ^12^, ^13^, ^14^, ^15^, ^16^ Only one of these was from North America.^12^

Before the advent of antibiotics, the most common cause of mycotic AAAs was infectious endocarditis. More common nowadays is hematogenous or lymphatic bacterial spread in individuals with risk factors such as arterial damage from iatrogenic manipulation, immunodeficiencies, and intravascular drug use.^17^ The most common comorbid condition for invasive GBS disease is diabetes mellitus (20%-25% of nonpregnant adults with GBS disease), in addition to chronic alcohol use, cirrhosis, cardiac disease, kidney disease, and neurological disorders.^6^^,^^7^ Interestingly, in our case, no source for the infection could be identified. Our patient was diabetic, but otherwise there were no risk factors for invasive GBS or mycotic aneurysms more generally.

Surgery is an essential component in the management of mycotic AAAs and may include ex situ or in situ repair. Potential disadvantages to ex situ repair include concerns of graft patency, as well as aortic stump rupture caused by residual tissue infection.^18^ In situ revascularization has gradually become the standard, owing to superior outcomes in the literature. In a study of 44 mycotic AAAs, the in-hospital mortality among those with in situ repair was 18.9% compared with 50% in those who underwent extra-anatomic reconstruction.^19^

When opting for in situ repair, there are several graft options. Prosthetics such as Dacron or polytetrafluoroethylene may be used, and despite impregnating with rifampin, the risk of graft infection must be taken into consideration. Other means of reducing infection risk may be employed including soft-tissue coverage with a pedicle of uninfected tissue such as the omentum.^20^ Autologous femoral vein grafts have excellent resistance to infection, but potential disadvantages include a long harvesting time, postoperative limb swelling, and graft occlusion due to valve-related thrombosis.^21^ Alternatively, cryopreserved arterial allografts may also offer superior resistance to abdominal aortic reinfection compared with prosthetic counterparts such as silver-coated Dacron grafts.^22^ However, disadvantages to this approach include lack of availability in urgent applications, as well as risk of late degeneration and thrombotic occlusions,^23^ although the latter seems to have improved with newer generations of the graft.

More recently, cryopreserved venous allografts have shown promise for their low rates of reinfection and reintervention. In a study of 23 suprainguinal arterial reconstructions using cryopreserved venous allografts—5 of which were mycotic AAAs—graft reinfection occurred in only one patient and two required reintervention for anastomotic dilatation.^24^ This is a similarly low rate of reinfection as found in the largest study of cryopreserved arterial allografts for aortic reconstruction, which reported reinfection in 8 of 220 (4%) cases.^25^ The use of cryopreserved vein in this setting remains uncommon; among 56 mycotic AAAs in a multicenter study of all cases across six countries from 2006 to 2016, only two procedures used cryopreserved venous allografts.^26^ In our patient, given its risk of reinfection, a prosthetic graft was not ideal and instead autologous or cryopreserved femoral vein was considered. Although there is insufficient data to compare the long-term patency of the two options, we opted for cryopreserved vein to avoid the morbidity associated with bilateral femoral vein harvest.

In our review of the literature, we did not find any previous cases of a cryopreserved venous allograft used in the repair of a mycotic AAA caused by GBS. Among the nine other reported cases, one used an iliac arterial homograft^16^ and another used an aortic allograft.^10^ Otherwise, one used an equine pericardial straight roll graft,^11^ and the remaining six used prosthetic grafts.^8^^,^^9^^,^^12^, ^13^, ^14^, ^15^

## Conclusions

Mycotic AAAs caused by GBS infection are rare with generally favorable surgical outcomes in previous reports. Cryopreserved femoral vein allograft may be an ideal choice for in situ repair to mitigate concerns of graft reinfection while negating the morbidity of femoral vein harvests. The long-term durability of cryopreserved femoral vein for aortic reconstruction remains to be determined.
